# Identification of a Diagnostic Set of Endomyocardial Biopsy microRNAs for Acute Cellular Rejection Diagnostics in Patients after Heart Transplantation Using Next-Generation Sequencing

**DOI:** 10.3390/cells8111400

**Published:** 2019-11-06

**Authors:** Tereza Nováková, Táňa Macháčková, Jan Novák, Petr Hude, Július Godava, Víta Žampachová, Jan Oppelt, Filip Zlámal, Petr Němec, Helena Bedáňová, Ondřej Slabý, Julie Bienertová-Vašků, Lenka Špinarová, Jan Krejčí

**Affiliations:** 1 Department of Cardiovascular Diseases, St. Anne’s University Hospital and Faculty of Medicine, Masaryk University, Pekařská 53, 65691 Brno, Czech Republic; 2 Central European Institute of Technology, Masaryk University, Kamenice 5, 62500 Brno, Czech Republic; 3 Department of Pathological Physiology, Masaryk University, Kamenice 5, 62500 Brno, Czech Republic; 4 Department of Pathology, St. Anne’s University Hospital and Faculty of Medicine, Masaryk University, Pekařská 53, 65691 Brno, Czech Republic; 5 Centre of Cardiovascular Surgery and Organ Transplantation, Pekařská 53, 65691 Brno, Czech Republic

**Keywords:** microRNA, endomyocardial biopsy, heart transplantation, acute cellular rejection, diagnostics

## Abstract

Introduction: Acute cellular rejection (ACR) of heart allografts represents the most common reason for graft failure. Endomyocardial biopsies (EMB) are still subject to substantial interobserver variability. Novel biomarkers enabling precise ACR diagnostics may decrease interobserver variability. We aimed to identify a specific subset of microRNAs reflecting the presence of ACR. Patients and Methods: Monocentric retrospective study. A total of 38 patients with the anamnesis of ACR were identified and for each patient three consecutive samples of EMB (with, prior and after ACR) were collected. Sixteen trios were used for next-generation sequencing (exploratory cohort); the resting 22 trios were used for validation with qRT-PCR (validation cohort). Statistical analysis was performed using R software. Results: The analysis of the exploration cohort provided the total of 11 miRNAs that were altered during ACR, the three of which (miR-144, miR-589 and miR-182) were further validated in the validation cohort. Using the levels of all 11 miRNAs and principal component analysis, an ACR score was created with the specificity of 91% and sensitivity of 68% for detecting the presence of ACR in the EMB sample. Conclusion: We identified a set of microRNAs altered in endomyocardial biopsies during ACR and using their relative levels we created a diagnostic score that can be used for ACR diagnosis.

## 1. Introduction

Heart failure (HF) is a clinical syndrome characterized by the presence of typical symptoms and signs of cardiac insufficiency (such as breathlessness or ankle oedema) [1]. HF affects approximately 1%–2% of the adult population and its prevalence rises over 17% in the elderly population over 85 years of age [1,2]. One-year all-cause mortality rates are 7% for patients with ambulatory HF and 17% for hospitalized HF; (re)-hospitalization rates are 32% and 44%, respectively, which make HF still one of the commonest causes for hospitalizations in the developed countries [3]. Despite the significant progress reached in the pharmacological and non-pharmacological therapies, orthotopic heart transplantation (OHT) remains one of the last options for patients with refractory HF whom still reside in the functional class NYHA III-IV, despite maximal therapy [1]. Overall survival and quality of life of patients after OHT, as compared with the natural course of end-stage HF, is outstanding and according to the International Society for Heart and Lung Transplantation (ISHLT) data, one year survival of patients after OHT is 84.5% and 5-year survival is 72.5% [4]. The causes of death vary over the time from OHT: graft failure is the leading cause of death within the first year, and in the long run, infections, allograft vasculopathy and renal failure take the lead. The most common cause of graft failure, especially in early postoperative period, is acute cellular rejection (ACR), i.e., infiltration and damage of myocardial tissue by immune cells. ACR represents one of the greatest threats for both transplanted patients and their care-taking physicians. The prevention and treatment of ACR is crucial not only because of the immediate risk of actual ACR leading to graft damage and causing symptoms of cardiac decompensation, but also because every single ACR episode predisposes for later development of graft failure and the development of cardiac allograft vasculopathy [5], as well as increasing the risk of death of transplanted patients [6]. The current “gold standard“ used already for more than 50 years in ACR diagnostics is the regular performance of endomyocardial biopsies (EMBs) [7] that are subsequently evaluated using histopathological examination based on the revised ISHLT scoring system [8]. However, the ISHLT grading system is still subject to substantial interobserver variability [9] and also some cases of biopsy-negative rejection have been described [10]. Thus, the identification of novel markers that would refine the diagnostics of ACR from EMBs is imperative that would further improve the quality of care for transplanted patients.

One of the novel promising groups of biomarkers in the field of cardiovascular medicine, and also in many other fields of medicine, is microRNAs (miRNAs, miRs). miRNAs are a group of small non-coding RNA that act intracellularly as the post-transcriptional gene expression regulators and extracellularly as novel tools for intercellular communication [11]. Their expression in tissues and extracellular fluid is known to be altered under various pathological conditions and both tissue and extracellular miRNAs have already proven to be promising markers in the field of oncology [12,13,14], neurodegenerative disorders (e.g., Alzheimer´s disease [15,16]) or in inflammatory disorders such as rheumatoid arthritis [17] or inflammatory-bowel disease [18]. In the field of cardiovascular medicine, it was reported that the tissue expression of miRNAs alters during coronary artery disease [19], heart failure [20] or atrial fibrillation [21]. Very recently, the changes in miRNA expression in EMBs were reported to occur during ACR as well as other types of rejection [22]. Within this study, we focused on ACR and we employed next-generation sequencing to identify a specific subset of miRNAs altered during ACR in EMBs in patients after OHT.

## 2. Materials and Methods

### 2.1. Study Population

The study was designed as a monocentric retrospective study and approved by the Multicentre Ethic Comittee of the St. Anne´s University Hospital (Number code: 1G/2016). Electronic medical records of patients after OHT that are followed by the Department of Cardiovascular Diseases of the St. Anne’s University hospital were searched automatically using the terms “orthotopic heart transplantation”, “OHT”, “IB”, “IB-II“, “III”, “0”, “1R”, “2R” or “3R”. The search period was between 01/2010 and 12/2015. Altogether, 84 patients fulfilling the search criteria were identified. Medical records of these patients were proofread by the cardiologist to confirm all identified patients indeed represent patients after OHT that experienced at least one episode of ACR. After proof-reading, 6 patients had to be excluded (their medical records contained searched words, however only in the context of clinical reasoning, and they did not have ACR). In the remaining 78 patients, histopathological electronic records (containing sample histopathological description and ISHLT grading) from all performed EMBs within the first year after OHT were identified and the list of potential candidates and wanted samples was created.

In each patient, we aimed to identify three distinct consecutive samples: sample with ongoing ACR of grade IB and higher (“rejection samples”) and two samples with no histological signs of rejection (i.e., ISHLT grade 0), one of them prior (i.e., “before rejection samples”) and one of them after (i.e., “after rejection samples”) ACR. In 35 patients, at least one of the abovementioned samples were missing (i.e., there was no sample prior or after the rejection with ISHLT grade 0) and in 5 patients we identified all wanted samples; however, available tissues samples were too small for sufficient RNA isolation and further processing, thus complete samples trios were in the end obtained from 38 patients. These samples were proofread by an experienced pathologist to confirm the presence of ACR and its grade. In the Czech Republic, the revised ISHLT grading system has been gradually accepted in the past 15 years, and usually both the original and revised gradings are still being used simultaneously; i.e., ACR is described by using the original grading (i.e., 0, IA, IB, II…) and revised grading (i.e., 0R, 1R, 2R…). In this study, the former grading was used as it was available and confirmed in all samples.

For the identification of novel microRNAs that would be suitable as novel biomarkers of ACR, we selected the two-step approach. First, within the exploratory phase of the study, we randomly selected 16 trios and performed next-generation sequencing (as described below) to identify all miRNAs present in the samples and to identify those altered during the ACR. Within the second, validation phase of the study, we used the remaining 22 patient trios and we determined the levels of statistically significantly dysregulated miRNAs identified in the exploratory phase using RT-qPCR (as described below).

**Figure 1:**
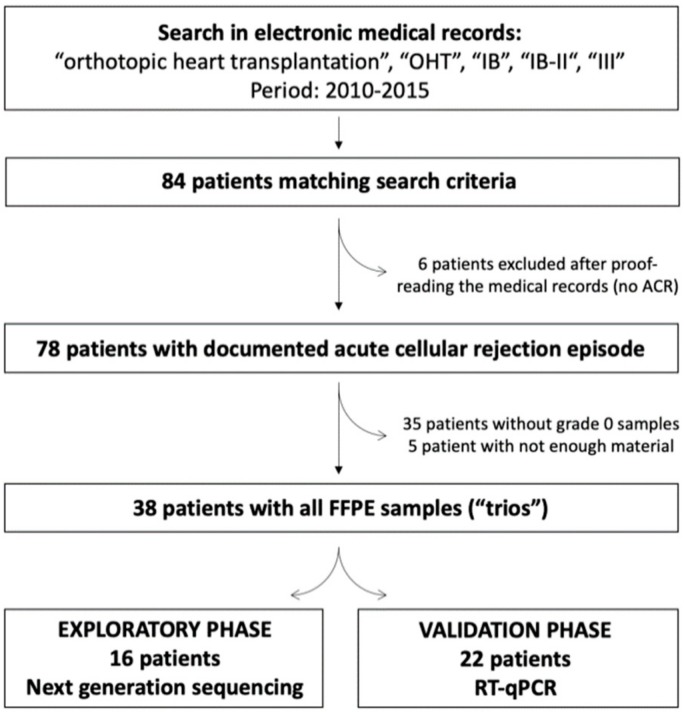
Study flowchart, summarizing the selection of patients for the final analysis. The deeper explanation is provided in the text.

The study flow is summarized in Figure 1 and the final study population (*n* = 38) characteristics are summarized in Table 1.

### 2.2. RNA Isolation

The total RNA enriched for a fraction of small RNAs was isolated from formalin-fixed paraffin-embedded (FFPE) samples of EMBs—10 FFPE slices with a thickness of 10 μm were used in a single isolation procedure. Samples first underwent xylene deparaffinization that was followed by RNA isolation using a mirVana™ miRNA isolation kit (Ambion, Thermo Fisher Scientific, Inc., Waltham, MA, USA) according to the manufacturer’s protocol. Fluorescence was then employed to determine total RNA concentration using a Qubit RNA HS Assay Kit and Qubit 2.0 Fluorometer (Invitrogen, Carlsbad, CA, USA)—the average RNA concentration was 3.41 ng/μL. No pooling of the samples was performed.

### 2.3. Exploratory Phase: Next-Generation Sequencing (NGS)

In total, 16 samples before rejection (BR), with rejection (R) and after rejection (AR) were randomly selected from the whole cohort for miRNA profiling using NGS. Thus, 48 samples were used for the preparation of cDNA sequencing libraries using a CleanTag^®^ Small RNA Library Prep Kit (Trilink, San Diego, CA, USA). Due to low RNA concentrations of the samples, 2 μL of undiluted eluate from the RNA isolation was used as input material. Adaptors were ligated to the 3′ and 5′ ends of miRNAs followed by reverse transcription. Subsequently, 21 cycles of PCR amplification with unique barcode-labelled amplification primers were performed, and purification was done using AMPure XP beads (Beckman-Coulter, Brea, CA, USA). The concentration and length of prepared cDNA libraries were measured using Agilent High sensitivity D1000 Screen Tape System and Agilent TapeStation System (Agilent Technologies, Santa Clara, CA, USA), and Qubit dsDNA HS Assay Kit (Invitrogen, Carlsbad, CA, USA) and a Qubit 2.0 Fluorometer (Invitrogen, Carlsbad, CA, USA). Equimolar amounts of each cDNA library were pooled at a final concentration of 4 nM, and samples were sequenced in a single-end read mode using NextSeq^®^ 500/550 High Output Kit v2 (75 cycles) chemistry (Illumina, San Diego, CA, USA) and Nextseq 500/550 machine (Illumina, San Diego, CA, USA). Count-based miRNA expression data were generated by the Chimira tool from fastq files. All sequences were adapter trimmed and mapped against miRBase v20 allowing up to two mismatches per sequence. Further analyses were performed using the R/Bioconductor packages edgeR and Deseq2.

### 2.4. Validation Phase: Reverse Transcription and Quantitative PCR (RT-qPCR)

Using microRNA-specific primers TaqMan MicroRNA Assay (Applied Biosystems, Foster City, CA, USA) and TaqMan MicroRNA Reverse Transcription Kit (Applied Biosystems, Foster City, CA, USA) according to manufacturer’s protocol, complementary DNA was synthesized from the total RNA enriched for a fraction of small RNAs.

Using the QuantStudio 12K Flex Real-Time PCR system (Applied Biosystems, Foster City, CA, USA), Universal PCR Master Mix (Applied Biosystems, Foster City, CA, USA), and TaqMan MicroRNA Assay (Applied Biosystems, Foster City, CA, USA) according to the manufacturers protocol, real-time PCR was performed (for details about used stem-loop primers and assays, see Supplementary Material 1). All real-time PCR reactions were run in duplicates and the average expression levels of all measured miRNAs were normalized using RNU48 and subsequently analysed by the 2−ΔCt methods.

### 2.5. Statistics

Statistical differences between the levels of analysed miRNAs in EMBs with, before and after ACR were evaluated by non-parametric Kruskal–Wallis tests. Paired samples before and during rejection or after and during rejection, were compared using the non-parametric Mann–Whitney test. The correlation among individual miRNAs was determined using the Pearson correlation coefficient. The modelling of the dependence of miRNA expression on time was performed using generalized least squares method. Calculations were performed using GraphPad Prism version 8.00 (GraphPad Software, La Jolla, CA, USA) and the R environment (R Development Core Team). *p* values < 0.05 were considered statistically significant.

## 3. Results

### 3.1. Small RNA Sequencing Reveals Several Sets of microRNAs Altered during ACR

**Figure 2:**
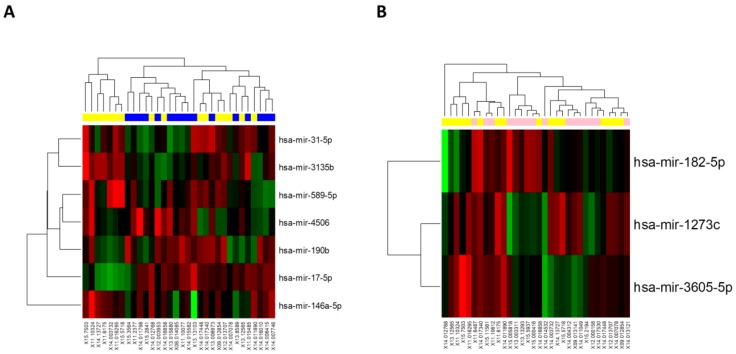
Hierarchical clustergram. Hierarchical clustergram discriminating miRNA expression in paired samples. Part **A** is based on 7 miRNAs comparing samples before rejection (shown in blue) and during rejection (shown in yellow). Part **B** is based on 3 miRNAs comparing samples during rejection (shown in yellow) and after rejection (shown in pink). All miRNAs were statistically significant and differentially expressed (*p* < 0.05). The gradient of green and red colours is used as the heatmap (green colour indicates lower expression whereas red colour indicates higher expression of individual miRNAs in analysed samples).

Using next-generation sequencing, we have identified 488 distinct miRNAs to be expressed in the EMB samples (data not shown). Comparison of relative expression of all identified miRNAs between samples with rejection and BR yielded seven miRNAs to be statistically significantly altered (hsa-miR-31-5p, hsa-miR-3135b, hsa-miR-589-5p, hsa-miR-4506, hsa-miR-190b, hsa-miR-17-5p, hsa-miR-146-5p, all <0.05) as shown in Figure 2A. Comparison of relative levels of all identified miRNAs between samples with R and AR yielded three miRNAs to be statistically significantly altered (hsa-miR-182-5p, hsa-miR-1273c, hsa-miR-3605-5p; *p* < 0.05) as shown in Figure 2B. After these individual comparisons, all miRNAs were also compared among all groups (BR, R and AR samples) and 11 miRNAs were showed to be statistically significantly dysregulated even after the adjustment for multiple comparisons (*p* < 0.05). Three different patterns were observed: Expression of six miRNAs was shown to increase during the rejection as compared with BR and AR samples (hsa-miR-3135b, hsa-miR-146a-5p, hsa-miR-589-5p, hsa-miR-1273c, hsa-miR-31-5 and hsa-miR-3605-5p), expression of three miRNAs was shown to decrease during the rejection as compared with BR and AR samples (hsa-miR-182-5p, hsa-miR-17-5p and hsa-miR-4506) and expression of two miRNAs were shown to increase during rejection as compared with the BR samples and to further increase in AR samples (hsa-miR-144-3p and hsa-miR-10b-5p). All of the miRNAs that have shown statistically significant altered expression among BR, R and AR samples were then selected for further validation.

### 3.2. Validation of Identified microRNAs Altered during ACR on the Validation Cohort

**Figure 3:**
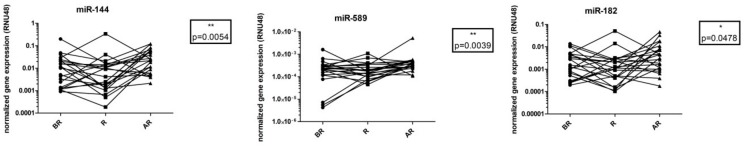
Validated miRNAs differentially expressed during acute cellular rejection (ACR). The figure represents the visualization of relative expression levels of three miRNAs, miR-144, miR-589 and miR-182, respectively among the samples before rejection (BR), during rejection (R) and after rejection (AR).

The expression levels of all 11 candidate miRNAs were determined in the validation cohort of 22 patients by RT-qPCR. Using Kruskall–Wallis comparison of three groups, relative expression levels of miR-144 (*p* = 0.0054), miR-589 (*p* = 0.0039) and miR-182 (*p* = 0.0478) were confirmed to be statistically significantly altered during the rejection (Figure 3). Differences in the relative expression levels of miR-31-5p were of borderline significance (*p* = 0.0885) and expression of all other miRNAs, i.e., miR-10b (*p* = 0.9600), miR-17 (*p* = 0.6994), miR-146a (*p* = 0.2956), miR-1273 (*p* = 0.9259), miR-3135b (*p* = 0.3994), miR-3605 (*p* = 0.5069) and miR-4506 (*p* = 0.9921) were not statistically significantly different. Relative expression levels of RNU48 did not differ among the groups (*p* = 0.1637).

### 3.3. Dependence of miRNA Levels on Time

Linear regression modelling was employed to study the dependence of miRNA levels on individual time points (i.e., before, during and after rejection). Using the generalized least squares method, expression of miR-144 (*p* = 0.008) and miR-589 (*p* = 0.002) showed to be statistically significantly dependent on time which is suggestive of their involvement in the rejection process.

### 3.4. Correlation of Individual microRNAs Expression at Different Time Points

Expression of individual miRNAs was separately correlated at the time before rejection, during rejection and after rejection. Numerous correlations were identified for individual miRNAs (Supplementary Table S1). Interestingly, the number of distinct correlations was highest during the rejection and some of the correlations were not observed in the samples prior to rejection and with rejection.

### 3.5. ROC Analysis, Regression Modelling and Principal Component Analysis Reveals a Score for ACR Diagnostics

For every individual miRNA, we created a logistic regression model based on categorizing the samples before rejection as “healthy controls” and patients with rejection as “cases”. For every model, the receiver operating curve (ROC) was created and using the Youden index, the cut-off value was selected, and sensitivity and specificity were determined (Supplementary Table S2). Within these models, individual miRNAs did not prove to be sensitive and specific enough to be used for ACR detection in an isolated manner.

Subsequently, we performed principal component analysis (PCA) to evaluate possible relationships between measured values. Levels of RNU48 and all miRNAs were logarithmically transformed and standardized to obtain standard distribution (Supplementary Table S3A). Principal components represent linear combinations of original values and coefficient’s describing these linear combinations are represented by values called “loadings” that in fact reflect the value of the correlation coefficient (Supplementary Table S3B). For individual principal components, logistic regression modelling was also performed and showed one model to be statistically significant and two being of borderline statistical significance (Supplementary Table S3C).

**Figure 4:**
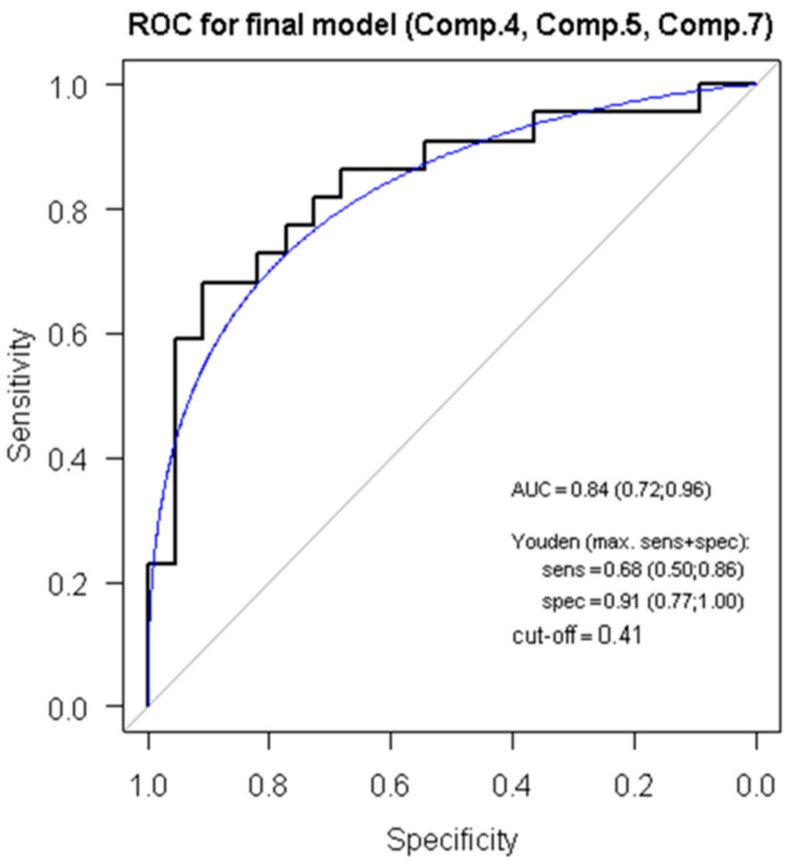
ROC analysis for selected principal components. ROC: Receiver operating characteristics analysis of the use of 3 main components (Comp. 4, Comp. 5 and Comp. 7) in discriminating between ACR and non-ACR samples. AUC = area under curve; Youden = Youden index; sens = sensitivity, and spec = specificity.

Using the stepwise backward regression method, we created a final model comprising the total of three of the principal components (Components 4, 5 and 7) and we obtained the ROC curve with AUC 0.84 (0.72; 0,96). Using the Youden index, the cut-off value of 0.408 was determined as a value with the highest specificity 0.91 (0.77; 1.00) and sensitivity 0.68 (0.50; 0.86) as shown in Figure 4.

This final model enables us to determine the ACR SCORE reflecting the presence or absence of ACR in the studied samples. Relative expression levels of all miRNAs and RNU48 obtained from the RT-qPCR shall be standardized and subsequently implemented into the following formula:

ACR SCORE = −0.055 – 0.464 × stand(log(miR144)) + 0.198 × stand(log(miR589)) + 1.304 × stand(log(miR146)) – 0.510 × stand(log(miR182)) – 0.227 × stand(log(miR3135b)) – 0.690 × stand(RNU48) – 0.065 × stand(log(miR10)) + 0.424 × stand(log(miR31)) – 0.196 × stand(log(miR17)) + 0.664 × stand(log(miR1273)) + 0.587 × stand(log(miR3605)) – 0.519 × stand(log(miR4506)).

The cut-off value for the ACR SCORE model is 0.408, as mentioned above. If the value obtained from the formula for the individual sample is below the cut-off, there is no ACR, if the value obtained from the formula for the subject is above the cut-off, ACR is present with the specificity of 91% and sensitivity of 68%.This section may be divided by subheadings. It should provide a concise and precise description of the experimental results, their interpretation as well as the experimental conclusions that can be drawn.

## 4. Discussion

ACR of cardiac allografts represents a highly relevant clinical condition that needs to be properly diagnosed and treated. As the precise identification and grading of a rejection episode results in the earlier tailoring and delivery of immunosuppression therapy, there is a lot of effort to search for markers of ACR as early as possible.

Within our study, we identified a set of miRNAs with the altered expression in EMBs that can be used to distinguish the samples with ACR from those without ACR with high sensitivity and specificity. This set of miRNAs may therefore be added to current ACR histopathological evaluation to further strengthen the diagnostic algorithm for ACR. In this study, we used the two-step approach and contrary to the previously published studies that used microarray profiling, we utilized next-generation sequencing (NGS). NGS was used in the early, exploratory phase on the cohort of 16 patients and led to identification of a set of 11 miRNAs that were statistically significantly dysregulated in EMB during the ACR when comparing the EMB samples obtained before and after the ACR. In the second step, this set was validated on an independent cohort of 22 individuals after OHT and three of the selected miRNAs (miR-144, miR-589 and miR-182) were confirmed to be statistically significantly dysregulated. The levels of hsa-miR-144 and hsa-miR-589 were shown to be dependent on time indicating their expression changes are indeed occurring as a result of ongoing ACR. Interestingly, we identified a high number of positive and negative correlations among expression levels of individual miRNAs, with most of them being present during the ongoing ACR—this may indicate that these miRNAs are involved in various pathological pathways that are all activated during the rejection process. Such observation further highlights the complexity of the whole ACR process including inflammatory response, cardiomyocytes apoptosis, necrosis, myocardial fibrosis and other processes as described further for individual miRNAs.

Several of the miRNAs that were identified by miRNA profiling in our study have been already associated with ACR in patients after OHT. Therefore, our results further support the plausibility of involvement in ACR process and the potential use of these miRNAs as ACR biomarkers. Notably, altered expression of miR-182 (the levels of which have been altered both in the exploratory and validation cohort) has already been reported in the very first report from 2012 focusing on the role of miRNAs in the pathophysiology of ACR. Wei et al. performed microarrays miRNA profiling in murine model of cardiac allograft rejection and miR-182 was found be increased in allograft tissues, in graft-infiltrating mononuclear cells and also in plasmas implicating its potential use in the ACR pathophysiology and monitoring [23]. Moreover, in their subsequent study, Wei et al. showed that the miR-182 originates mainly from the CD4+ T-cells and that the absence of miR-182 in studied animals (homozygous miR-182 deficient mice) augmented allograft survival suggesting that miR-182 could also serve as a potential therapeutic target [24]. miR-182 is a part of the cluster consisting of miR-183/-96/-182 that is highly conserved across species [25] and is known to regulate expression of FOXO1 [23,24]. FOXO1 is a crucial regulator of a variety of cellular processes, including apoptosis, proliferation and cell survival, and is also involved in maintaining homeostasis of T- and B-lymphocytes and neutrophils [23,24]. Another miRNA with significant implications for ACR from our study, miR-144, was previously shown to regulate the transforming growth factor β (TGFβ) signalling cascade, another of the important signalling cascades in the immune cells´ homeostasis and also in cardiac allograft fibrosis development [26]. MiR-144 is also part of the inflammatory response via promoting production of the pro-inflammatory cytokines through regulation of ATB-binding cassette A1 (ABCA1) expression and cholesterol homeostasis [27]. It has to be mentioned, however, that miR-144 has been repeatedly reported to be specifically expressed in the red blood cells and it is believed to have biased the results of many studies as its targets may not be biologically relevant [28]. Thus, the changes in the levels of this miRNA during ACR may not reflect the changes occurring in the myocardium but rather the changes occurring directly in the red blood cells or may reflect rather the number of the red blood cells present in myocardium. In accordance with this reasoning, the recent study by Di Francesco et al. [22] highlights the importance of the cellular origin of individual miRNAs and the type of the ongoing rejection; using miRNA profiling, the authors identified miRNA profiles specific for ACR, but also for antibody-mediated rejection (AMR) and mixed rejection. By using in situ PCR expression, the authors of this study showed that two of the studied miRNAs, miR-29b-3p and miR-126-5p, were differentially expressed in one tissue during ACR (miR-126-5p to be overexpressed in endothelial cells and miR-29b-3p to be overexpressed in fibroblasts) and in another tissue during AMR (miR-126-5p to be overexpressed in cardiomyocytes and miR-29b-3p to be underexpressed and present only in cardiomyocytes) [22]. Nevertheless, even though the biological role of the miRNA may not be directly related to the rejection process, its potential to be used as a clinically relevant biomarker per se still should be evaluated.

In contrast to our results, there are also other miRNAs that were repeatedly reported by other research groups to be associated or involved in the ACR process, but their levels were not validated in our study, such as miR-146a (altered expression of miR-146a was observed only in the exploratory phase and was not confirmed in the validation phase) or miR-155. Both miR-146a and miR-155 were shown to be increased in animal models of cardiac mouse-to-rat xenotransplantation [29]. Furthermore, in 2016, Van Aelst et al. performed both miRNA and mRNA profiling from human and mouse samples of cardiac and renal ACR to identify common, non-organ-specific markers of ACR [30]. In the subset of cardiac ACR, they identified 10 dysregulated miRNAs (miR-21/-142-3p/-142-5p/-146a/-146b/-149-5p/-155/-222/-223/-494), and both miR-146a and miR-155 changes were observed, with miR-155 levels being the most dysregulated among all studied miRNAs. The authors highlighted the role of miR-155 in the regulation of SPI1 (spleen focus forming virus proviral integration oncogene), which is a transcription factor involved in interleukin-6 expression regulation [30]. The functional studies utilizing miR-155 inhibition either by antagomiR-155 (i.e., application of oligonucleotide sequence targeted against miR-155 causing its blockade) [30] or using miR-155 knockout animals [31] also showed improvement of the allograft function and amelioration of ACR course, which further highlights the importance of this miRNA in ACR process.

Furthermore, some of the miRNAs identified in our study have never been described in the context of ACR of cardiac allografts and in some of them, their biological role still needs to be elucidated. Hsa-miR-10b-5p was shown to be increased in plasma of patients with hypertrophic cardiomyopathy in whom it was associated with the presence of myocardial fibrosis determined by cardiac magnetic resonance [32]. hsa-miR-17-5p is part of the miR-17-92 cluster that is well known for its link to tumorigenesis [33]; however, several functions in the myocardium have already been described including regulation of myocardial fibrosis [32] or cardiomyocytes apoptosis [34,35]. Just recently, its role in predicting heart failure development after myocardial infarction was reported [36]. The circulating levels of hsa-miR-31-5p were shown to be increased in patients with coronary artery disease [37]. Increased plasmatic levels of hsa-miR-3135b were observed in patients with dilated cardiomyopathy [38] and heart failure [39]. The functional roles of another identified miRNA have so far been described only in the context of tumorigenesis of various cancers (as for hsa-miR-589 [40,41] or hsa-miR-1273c [42]) and the biological function of the rest of the identified miRNAs (hsa-miR-3605-5p, has-miR-4506) is currently unknown.

In general, there are several possible explanations for the heterogeneity of results in the studies that were performed so far. The individual studies differ significantly in methodology—the studies involving human subjects are using EMB samples obtained from the right ventricle, while the preclinical studies based on the cardiac allografts of experimented animals usually use the heart as a whole (i.e., including myocardium from left and right ventricles and atria). Several reports indicate that miRNA expression differs in individual heart chambers (e.g., between left and right ventricles or atria [43]) and this may significantly affect the results. In contrast to the results of our study, most of the previously published studies were using microarray profiling, while in our study we used small RNA sequencing—both methods have their specific advantages and limitations. Another source of variability may arise from the different RNA isolation protocols according to different manufacturers. Beyond the methodology, as we mentioned above, ACR is a highly complex process and includes several different sub-processes that are occurring at the same time (e.g., infiltration by leukocytes, increase in vascular permeability, fibrosis, apoptosis or necrosis of cardiomyocytes, etc.) and it can be expected that the ratio among individual processes will be dependent on the interval from OHT to ACR and it will also be affected by used immunosuppressive strategy. Individual EMBs obtained from individual patients can also differ in the ratio of myocardial and other tissues (such as fibrotic tissue, vessels, and the amount of infiltrating leukocytes or red blood cells), which then affects miRNAs expression profiles shifting it closer to the dominating tissue in the sample (as documented, e.g., for miR-144 that is abundant in red blood cells [28]). Last but not least, the original underlying reason for OHT (e.g., observed differences between patients with coronary artery disease or dilated cardiomyopathy), the presence of other comorbidities (especially those associated with any kind of inflammation) or differences in applied immunosuppressive therapy may represent a significant source of variability.

Our study is unique in its approach by using small RNA sequencing, which allowed us to identify novel miRNAs that have never been associated with the rejection so far. This is so far the first study to perform principal component analysis in the settings of ACR diagnostics that allowed us to identify otherwise latent relationships among expression levels of studied miRNAs. The major contribution of the study is the creation of a clinically easily applicable ACR SCORE model requiring simply the measurement of levels of 11 identified miRNA and their internal control and filling obtained values into the formula to obtain a highly sensitive and specific value of an ACR SCORE, confirming or ruling-out the ACR.

There are several limitations to our study, mainly due to its retrospective design and the low abundance of ACR in the current era of modern immunosuppressive agents. Despite involvement of the total of 38 individuals, this study cohort is still too small to allow for the generalization of our results. The retrospective approach did not allow us to collect plasma samples and to study the correlation between the plasmatic and tissue miRNA expression, which we plan to investigate in our further ongoing studies and which we do believe is the key to enable non-invasive ACR diagnostics that would relief the patients from undertaking EMBs, thus increasing their safety and the quality of life. One of the limitations is also the fact that other types of rejection (such as AMR) are not addressed, mainly due to their low prevalence in our study cohort and also due to the lack of bioptic material. When performing the RNA isolation, we were working with the remnants of the EMB samples that had already been used for histopathological evaluation and obtained amount of isolated RNA was just sufficient to perform the planned analysis. Due to the low quantity of RNA it was not, therefore, possible to determine the levels of more miRNAs (e.g., those previously identified by other research teams), or to revalidate the expression of miRNAs identified by next-generation sequencing.

## 5. Conclusions

In this study, we have identified several miRNAs that were altered in the EMB samples from the patients with OHT suffering from the ACR. Using expression levels of all these miRNAs, we have created an ACR SCORE model using logistic regression that makes it possible to recognize ACR with high specificity and sensitivity. Further studies validating results of our study on larger cohorts of patients are needed to select the most sensitive and specific set of miRNAs and to implement miRNA use into the clinical practice.

## Figures and Tables

**Table 1:** Study cohort characteristics.

Parameter	Mean	SD	Median	Min	Max
Age at OHT (years)	53	10.6	58	21	65
Weight (kg)	86.2	17.2	84	56	124
Height (cm)	177.5	8.66	179	162	195
BMI (kg/m^2^)	27.19	4.01	26.84	17.28	37.12
Males/females	35/3				
Diagnosis	Dilated cardiomyopathy (19 patients) Ischemic heart disease (10 patients) Others * (9 patients)				
Immunosuppression	Cyclosporine (10.5% = 4 patients) Tacrolimus (26.3% = 10 patients) Everolimus (5.3% = 2 patients) Sirolimus (2.6% = 1 patient) Cyclosporine + MPA (2.6% = 1 patient) Tacrolismus + Everolimus (18.6% = 7 patients) Tacrolimus + MPA (28.9% = 11 patients) Everolimus + MPA (2.6% = 1 patient) Sirolimus + MPA (2.6% = 1 patient)				
Basic laboratory results (at the time of rejection)					
Urea (mmol/L)	9.532	4.00	8.65	3.6	24.9
Creatinine (μmol/L)	117.79	38.63	113.5	66	221
GFR (mL/min/m^2^)	1.03	0.34	0.97	0.50	1.76
AST (μkat/L)	0.48	0.21	0.45	0.16	1.34
ALT (μkat/L)	0.65	0.60	0.49	0.17	3.84
CRP (mg/L)	8.27	12.06	2.70	0.50	48.30
Erythrocytes (×10^12^/L)	4.42	0.66	4.29	3.20	5.99
Leukocytes (×10^9^/L)	7.70	3.24	7.15	2.80	19.60
Hemoglobin (g/L)	126.38	16.48	128.50	89.00	173.00
Hematocrit(L/L)	0.38	0.05	0.39	0.27	0.50
Thrombocytes (×10^9^/L)	201.71	59.04	209.50	93.00	305.00

Abbreviations: SD = standard deviation; OHT = orthotopic heart transplantation; GFR = glomerular filtration rate; CRP = C-reactive protein; MPA = mycophenolic acid. * Other causes included restrictive cardiomyopathy (2), HF post-childhood surgery (3: 1 × Fallot pentalogy, 1 × vessel transposition, 1 × Marfan’s syndrome after valve replacement), inflammatory cardiomyopathy (2), DCM+IHD (1), and AL-amyloidosis (1).

## Supplementary Materials

The following are available online at https://www.mdpi.com/2073-4409/8/11/1400/s1, Supplementary Material 1: TaqMan stem-loop primers and mature miRNAs sequences; Supplementary Table S1: Correlations among individual miRNAs; Supplementary Table S2: ROC analysis for individual miRNAs; Supplementary Table S3: Principal component analysis workflow. Sequencing data are available at the authors upon request.
