# Supplementary material for: Identification of a Diagnostic Set of Endomyocardial Biopsy microRNAs for Acute Cellular Rejection Diagnostics in Patients after Heart Transplantation Using Next-Generation Sequencing
Source: Cells. 2019 Nov 6;8(11):1400. doi: 10.3390/cells8111400 (PMC6912472; doi:10.3390/cells8111400)
Supplement: Supplementary file 1 [file cells-08-01400-s001.pdf]

Supplementary Material 1: TaqMan stem-loop primers and mature miRNAs sequences

hsa-mir-10b-5p

- assay ID: 002218
- Mature miRNA Sequence: UACCCUGUAGAACCGAAUUUGUG
- Stem-loop Accession number: MI0000267
- Stem-loop Sequence
  CCAGAGGUUGUAACGUUGUCUAUAUAUACCCUGUAGAACCGAAUUUGUGUGGUAUCCGUAUAGUCACAGAUU CGAUUCUAGGGGAAUAUAUGGUCGAUGCAAAAACUUCA

hsa-mir-17-5p

- assay ID: 002308
- Mature miRNA Sequence: CAAAGUGCUUACAGUGCAGGUAG
- Stem-loop Accession number: MI0000071
- Stem-loop Sequence
  GUCAGAAUAAUGUCAAAGUGCUUACAGUGCAGGUAGUGAUAUGUGCAUCUACUGCAGUGAAGGCACUUGUAG CAUUAUGGUGAC

hsa-mir-31-5p

- assay ID: 002279
- Mature miRNA Sequence: AGGCAAGAUGCUGGCAUAGCU
- Stem-loop Accession number: MI0000089
- Stem-loop Sequence
  GGAGAGGAGGCAAGAUGCUGGCAUAGCUGUUGAACUGGGAACCUGCUAUGCCAACAUAUUGCCAUCUUUCC

hsa-mir-144-3p

- assay ID: 002676
- Mature miRNA Sequence: UACAGUAUAGAUGAUGUACU
- Stem-loop Accession number: MI0000460
- Stem-loop Sequence
  UGGGGCCCUGGCUGGGAUAUCAUCAUAUACUGUAAGUUUGCGAUGAGACACUACAGUAUAGAUGAUGUACUA GUCCGGGCACCCCC

hsa-mir-146a-5p

- assay ID: 000468
- Mature miRNA Sequence: UGAGAACUGAAUUCCAUGGGUU
- Stem-loop Accession number: MI0000477
- Stem-loop Sequence
  CCGAUGUGUAUCCUCAGCUUUGAGAACUGAAUUCCAUGGGUUGUGUCAGUGUCAGACCUCUGAAAUUCAGUU CUUCAGCUGGGAUAUCUCUGUCAUCGU

hsa-mir-182-5p

- assay ID: 002334
- mature miRNA sequence: UUUGGCAAUGGUAGAACUCACACU
- Stem-loop Accession number: MI0000272
- Stem-loop Sequence
  GAGCUGCUUGCCUCCCCCCGUUUUUGGCAAUGGUAGAACUCACACUGGUGAGGUAACAGGAUCCGGUGGUUC UAGACUUGCCAACUAUGGGGCGAGGACUCAGCCGGCAC

hsa-mir-589-5p

- assay ID: 002409
- Mature miRNA Sequence: UGAGAACCACGUCUGCUCUGAG
- Stem-loop Accession number: MI0003599
- Stem-loop Sequence
  UCCAGCCUGUGCCCAGCAGCCCCUGAGAACCACGUCUGCUCUGAGCUGGGUACUGCCUGUUCAGAACAAAUGCC GGUUCCCAGACGCUGCCAGCUGGCC

hsa-mir-1273c

- assay ID: 243951_mat
- Mature miRNA Sequence: GGCGACAAAACGAGACCCUGUC
- Stem-loop Accession number: MI0014171
- Stem-loop Sequence
  UGCAGCCUGGGCGACAAAACGAGACCCUGUCUUUUUUUUUUUCUGAGACAGAGUCUCGUUCUGUUGCCCAAG CUGGA

hsa-mir-3135b

- assay ID: 464863_mat
- Mature miRNA Sequence: GGCUGGAGCGAGUGCAGUGGUG
- Stem-loop Accession number: MI0016809
- Stem-loop Sequence:
  UGCCCAGGCUGGAGCGAGUGCAGUGGUGCAGUCAGUCCUAGCUCACUGCAGCCUCGAACUCCUGGGCU

hsa-mir-3605-5p

- assay ID: 463752_mat
- Mature miRNA Sequence: UGAGGAUGGAUAGCAAGGAAGCC
- Stem-loop Accession number: MI0015995
- Stem-loop Sequence
  ACUUUAUACGUGUAAUUGUGAUGAGGAUGGAUAGCAAGGAAGCCGCUCCCACCUGACCCUCACGGCCUCCGUG UUACCUGUCCUCUAGGUGGGACGCUCG

hsa-mir-4506

- assay ID: 464024_mat
- Mature miRNA Sequence: AAAUGGGUGGUCUGAGGCAA
- Stem-loop Accession number: MI0016869
- Stem-loop Sequence
  UGGCCUCUGCCAUCAGACCAUCUGGGUUCAAGUUUGGCUCCAUCUUUAUGAAAUGGGUGGUCUGAGGCAAGU GGUCU

Supplementary Tables 1: Correlations among individual miRNAs

Correlations among individual miRNAs expression

Before rejection

Correlation coefficients

	miR-144	miR-589	miR-146	miR-182	miR-3135	miR-10	miR-31	miR-17	miR-1273	miR-3605
miR-589	0,50									
miR-146	0,80	0,76								
miR-182	0,85	0,64	0,74							
miR-3135b	0,06	0,50	0,30	0,20						
miR-10	0,57	0,60	0,58	0,64	0,30					
miR-31	-0,06	0,32	0,13	-0,13	0,24	0,11				
miR-17	0,58	0,61	0,57	0,73	0,25	0,68	-0,08			
miR-1273	0,28	0,42	0,29	0,31	0,19	0,71	0,24	0,49		
miR-3605	-0,11	-0,14	-0,23	0,03	-0,14	0,03	-0,14	0,09	0,42	
miR-4506	-0,08	-0,07	-0,20	0,04	0,28	0,16	-0,02	0,07	0,29	0,34

p-values

	miR-144	miR-589	miR-146	miR-182	miR-3135	miR-10	miR-31	miR-17	miR-1273	miR-3605
m589.norm.BR	0,018									
m146.norm.BR	<0,001	<0,001								
m182.norm.BR	<0,001	0,001	<0,001							
m3135.norm.BR	0,788	0,019	0,174	0,363						
m10.norm.BR	0,006	0,003	0,004	0,001	0,183					
m31.norm.BR	0,778	0,144	0,560	0,575	0,287	0,615				
m17.norm.BR	0,004	0,003	0,006	<0,001	0,264	<0,001	0,729			
m1273.norm.BR	0,213	0,049	0,195	0,155	0,398	<0,001	0,272	0,020		
m3605.norm.BR	0,616	0,520	0,308	0,909	0,527	0,885	0,544	0,698	0,050	
m4506.norm.BR	0,717	0,760	0,385	0,877	0,219	0,497	0,917	0,772	0,209	0,138

During rejection

korelační koeficienty

	miR-144	miR-589	miR-146	miR-182	miR-3135	miR-10	miR-31	miR-17	miR-1273	miR-3605
miR-589	0,73									
miR-146	0,45	0,22								
miR-182	0,94	0,76	0,44							
miR-3135b	0,56	0,49	0,40	0,49						
miR-10	0,58	0,60	0,24	0,59	0,34					
miR-31	0,19	0,38	0,02	0,26	0,24	0,55				
miR-17	0,82	0,79	0,33	0,81	0,52	0,71	0,47			
miR-1273	0,52	0,57	0,11	0,43	0,59	0,54	0,53	0,72		
miR-3605	0,61	0,67	-0,03	0,53	0,33	0,64	0,41	0,73	0,78	
miR-4506	0,39	0,52	0,25	0,34	0,56	0,72	0,42	0,52	0,62	0,41

	miR-144	miR-589	miR-146	miR-182	miR-3135	miR-10	miR-31	miR-17	miR-1273	miR-3605
miR-589	<0,001									
miR-146	0,035	0,323								
miR-182	<0,001	<0,001	0,039							
miR-3135b	0,006	0,019	0,062	0,022						
miR-10	0,005	0,003	0,292	0,004	0,126					
miR-31	0,410	0,078	0,917	0,233	0,276	0,008				
miR-17	<0,001	<0,001	0,140	<0,001	0,014	<0,001	0,026			
miR-1273	0,014	0,006	0,627	0,048	0,003	0,009	0,011	<0,001		
miR-3605	0,002	<0,001	0,883	0,011	0,130	0,001	0,058	<0,001	<0,001	
miR-4506	0,074	0,013	0,256	0,126	0,007	<0,001	0,054	0,013	0,002	0,060

After rejection

korelační koeficienty

	miR-144	miR-589	miR-146	miR-182	miR-3135	miR-10	miR-31	miR-17	miR-1273	miR-3605
miR-589	0,14									
miR-146	0,43	0,37								
miR-182	0,74	0,22	0,44							
miR-3135b	-0,16	0,32	0,22	-0,01						
miR-10	0,35	0,32	0,38	0,43	0,10					
miR-31	0,15	0,26	0,10	-0,08	-0,01	0,07				
miR-17	0,52	0,00	0,53	0,61	0,19	0,20	-0,09			
miR-1273	0,32	0,11	0,08	0,04	0,24	0,12	0,27	0,33		
miR-3605	-0,01	-0,18	0,14	0,08	0,01	0,13	-0,06	0,20	0,33	
miR-4506	-0,06	0,02	0,09	-0,18	-0,20	0,26	0,00	-0,01	0,26	0,43

	miR-144	miR-589	miR-146	miR-182	miR-3135	miR-10	miR-31	miR-17	miR-1273	miR-3605
miR-589	0,548									
miR-146	0,044	0,093								
miR-182	<0,001	0,335	0,043							
miR-3135b	0,477	0,144	0,328	0,982						
miR-10	0,108	0,152	0,082	0,044	0,643					
miR-31	0,501	0,246	0,664	0,711	0,973	0,749				
miR-17	0,013	0,993	0,010	0,003	0,394	0,381	0,686			
miR-1273	0,147	0,624	0,739	0,876	0,278	0,583	0,218	0,135		
miR-3605	0,980	0,424	0,542	0,714	0,953	0,567	0,805	0,373	0,133	
miR-4506	0,797	0,934	0,694	0,414	0,378	0,243	0,999	0,969	0,251	0,044

Supplementary Tables 2: ROC analysis for individual miRNAs

	β_0 estimate	β_1 estimate	OR estimate	p
miR-144	-1,147	-0,226	0,798	0,246
miR-589	0,849	0,098	1,103	0,708
miR-146	0,964	0,418	1,519	0,290
miR-182	-1,612	-0,245	0,783	0,273
miR-3135b	-0,092	-0,019	0,981	0,970
miR-10	0,097	0,018	1,018	0,944
miR-31	1,862	0,398	1,490	0,167
miR-17	0,063	0,099	1,104	0,790
miR-1273	3,225	0,414	1,513	0,242
miR-3605	4,620	0,530	1,699	0,134
miR-4506	-0,537	-0,112	0,894	0,652

Supplementary Tables 3: Principal component analysis workflow

A: Descriptive statistics

All miRNA levels were standardized. Levels of miRNAs, but not of RNU48, had to be first logarithmically transformed (marked with *)

	mean	SD
stand(miR144*)	-5,07	1,63
stand(miR589*)	-8,68	1,18
stand(miR146*)	-2,31	0,81
stand(miR182*)	-6,58	1,42
stand(miR3135*)	-4,83	0,61
stand(RNU48)	26,26	1,50
stand(miR10*)	-5,36	1,17
stand(miR31*)	-4,66	1,14
stand(miR17*)	-0,64	0,83
stand(miR1273*)	-7,78	0,94
stand(miR3605*)	-8,73	1,00
stand(miR4506*)	-4,79	1,26

B: Loadings representing coefficients of linear combinations for all principal components

	Principal components											
	Comp.1	Comp.2	Comp.3	Comp.4	Comp.5	Comp.6	Comp.7	Comp.8	Comp.9	Comp.10	Comp.11	Comp.12
stand(miR144*)	0,35	0,29	0,15	0,38	0,06	0,17	0,04	0,12	0,00	0,03	0,24	0,72
stand(miR589*)	0,32	0,20	-0,17	-0,29	-0,26	0,06	-0,30	-0,35	-0,65	-0,16	-0,04	0,06
stand(miR146*)	0,25	0,42	-0,19	-0,18	-0,06	-0,11	0,78	0,14	-0,03	-0,06	-0,15	-0,14
stand(miR182*)	0,35	0,32	0,22	0,12	0,02	0,03	-0,26	0,33	-0,02	0,14	0,40	-0,59
stand(miR3135*)	0,25	-0,01	-0,34	-0,34	0,53	0,49	-0,12	0,01	0,18	0,32	-0,14	0,02
stand(RNU48)	0,22	-0,23	-0,25	0,73	-0,02	0,27	0,13	-0,22	-0,13	-0,02	-0,27	-0,29
stand(miR10*)	0,36	-0,07	0,03	0,03	-0,08	-0,59	-0,08	-0,26	0,15	0,61	-0,19	0,06
stand(miR31*)	0,17	-0,35	-0,51	-0,07	-0,50	-0,01	-0,09	0,53	0,09	0,03	0,13	0,11
stand(miR17*)	0,38	0,05	0,21	-0,07	-0,07	-0,04	-0,26	0,08	0,44	-0,53	-0,50	-0,01
stand(miR1273*)	0,32	-0,35	0,11	-0,19	-0,12	0,14	0,25	-0,47	0,30	-0,17	0,53	-0,06
stand(miR3605*)	0,16	-0,41	0,60	-0,17	-0,11	0,27	0,22	0,26	-0,33	0,22	-0,25	0,02
stand(miR4506*)	0,24	-0,35	-0,09	0,02	0,59	-0,45	0,04	0,20	-0,31	-0,32	0,13	0,03

C: ROC analysis for individual principal components

	β_0 estimate	β_1 estimate	OR estimate	p
Comp.1	0,000001	0,017	1,017	0,898
Comp.2	0,002239	-0,246	0,782	0,309
Comp.3	0,000000	-0,001	0,999	0,998
Comp.4	-0,018466	-0,771	0,462	0,042
Comp.5	0,001039	-0,586	0,557	0,103
Comp.6	0,000022	0,081	1,085	0,842
Comp.7	0,005040	0,939	2,556	0,064
Comp.8	-0,000027	0,175	1,191	0,729
Comp.9	-0,000003	0,091	1,096	0,875
Comp.10	0,000006	-0,120	0,887	0,855
Comp.11	-0,001641	-0,909	0,403	0,266
Comp.12	0,000016	0,491	1,634	0,788
